# Insulin Resistance Markers to Detect Nonalcoholic Fatty Liver Disease in a Male Hispanic Population

**DOI:** 10.1155/2022/1782221

**Published:** 2022-08-03

**Authors:** Maritza Pérez-Mayorga, Jose P. Lopez-Lopez, Maria A. Chacon-Manosalva, Maria G Castillo, Johanna Otero, Daniel Martinez-Bello, Diego Gomez-Arbelaez, Daniel D. Cohen, Patricio Lopez-Jaramillo

**Affiliations:** ^1^Instituto Masira, Universidad de Santander (UDES), Bucaramanga, Colombia; ^2^Universidad Militar Nueva Granada, Bogotá, Colombia; ^3^Asociación Colombiana de Endocrinología, Diabetes y Metabolismo, Bogotá, Colombia; ^4^Hospital Universitario San Ignacio, Pontificia Universidad Javeriana, Bogotá, Colombia; ^5^Fundación Oftalmológica de Santander (FOSCAL), División de Investigaciones, Bucaramanga, Colombia; ^6^Universidad de, Buenos Aires, Argentina

## Abstract

**Background:**

Nonalcoholic fatty liver disease (NAFLD) is one of the leading causes of chronic liver disease and is closely associated with cardiometabolic disorders, being insulin resistance (IR) the common pathogenic mechanism. The triglycerides/glucose (TyG) index and triglycerides/HDL-c (TG/HDL) ratio are markers correlated with IR. We compared the capacity of these two indexes, alongside IR, to detect NAFLD.

**Methods:**

In a cross-sectional cohort study, we examined 263 active military personnel from the Colombian Air Force, aged between 29 and 54 years. Anthropometric measurements and biochemical determinations (glycemia, lipid profile, and insulin) were obtained, and ultrasound studies were performed to evaluate the presence of NAFLD. HOMA-IR index was calculated as (fasting insulin (*µ*IU/mL) × fasting glucose (mmol/L)/22.5), the TyG index as Ln (triglycerides (mg/dL) × fasting glucose (mg/dL)/2), and the TG/HDL ratio as (triglycerides (mg/dL)/HDL-c (mg/dL)).

**Results:**

NAFLD ultrasound criteria were met in 70 individuals (26.6%). Subjects with NAFLD had significantly higher values of HOMA-IR (2.55 ± 1.36 vs. 1.51 ± 0.91), TyG (9.17 ± 0.53 vs. 8.7 ± 0.51), and TG/HDL (6.6 ± 4.54 vs. 3.52 ± 2.32) compared to those without NAFLD (*p* < 0.001). A TyG cutoff point of 8.92 showed an AUC of 0.731, while cutoff points of 3.83 for TG/HDL and 1.68 for HOMA-IR showed an AUC of 0.766 and 0.781, respectively.

**Conclusion:**

Our study shows that novel and lower-cost markers of IR are useful for detecting NALFD, with a performance comparable to the HOMA-IR index. These markers should be used as the first step when screening patients for NAFLD.

## 1. Introduction

Nonalcoholic fatty liver disease (NAFLD) is defined as the presence of steatosis in at least 5% of the total hepatocytes in the absence of other recognizable causes (alcohol, viral, and autoimmune) [1]. It is estimated a global prevalence of approximately 25%, being higher in the Hispanic population and obesity and diabetes [2]. NAFLD is one of the most common liver diseases and a leading cause of chronic liver disease and an increasing indication of liver transplantation [3, 4]. Moreover, NAFLD is recognized as an independent risk factor for cardiovascular disease, and it is associated with cardiometabolic disorders such as dyslipidemia, type 2 diabetes, hypertension, and chronic kidney disease [5–8], so the term metabolic-associated fatty liver disease (MAFLD) has been proposed [9]. The common mechanism involved in developing NAFLD and cardiometabolic disease is insulin resistance (IR), which is initially triggered by visceral adipose tissue accumulation [10, 11]. Since NAFLD generates a high disease burden, increasing health services costs, its identification, and early management are necessary.

The Homeostasis Model Assessment index (HOMA-IR) has been the most commonly used method to detect IR because of its good correlation with the gold standard, the hyperinsulinemic-euglycemic clamp [12]. However, the HOMA-IR requires measuring insulin levels that usually have wide variability, mostly due to a lack of standardized insulin assays [13]. Also, measuring insulin levels is costly, making it often unaffordable at the primary care level, particularly in healthcare systems with lower financial resources, as in low-middle income countries. For example, in Colombia, the cost of a HOMA-IR measurement is approximately 2-3 times higher than a triglycerides/glucose measurement. Therefore, new indexes to assess IR indirectly have been proposed. These are based on the most frequent metabolic alterations associated with IR, such as high triglyceride levels, low HDL-c levels, and dysglycemia. The triglycerides/glucose index (TyG) and triglycerides/HDL-c ratio (TG/HDL) have emerged as the most promising means to easily detect IR and have demonstrated similar or even better performance to detect IR and its associated metabolic alterations compared to HOMA-IR. In addition, a positive association between TyG index and NAFLD or with hepatic steatosis severity and the presence of liver fibrosis has been demonstrated [14, 15]. However, most of this research has been conducted in Asian populations [16–25] with few studies conducted in Latin American populations [26, 27]. The present study aimed to compare the capacity of HOMA-IR, TyG, and TG/HDL to detect subjects with an ultrasound diagnosis of NAFLD in a sample of Colombian adults.

## 2. Materials and Methods

### 2.1. Participants

In a cross-sectional cohort study, we examined 263 active military personnel from the Colombian Air Force, aged between 29 and 54 years, who attended an annual preventive medical evaluation in 2006. The study was approved by the Research Division and the Ethics Committee of the Universidad Militar Nueva Granada, Bogotá, Colombia. Before any data collection or procedure was carried out, informed consent was obtained. Patients with acute diseases, chronic inflammatory diseases, and cancer were excluded.

### 2.2. Procedures

All participants were interviewed verbally to assess their cardiovascular risk factors and medication use. Anthropometric measurements were taken wearing light clothing and without shoes. Weight was measured on a scale and recorded to the nearest one-hundredth of a gram. Height was measured with a precision of 0.5 cm. Body mass index (BMI) was calculated as weight (kg) divided by the square of height (m). Waist circumference was measured in a standing position, placing a tape measure parallel to the floor at the midpoint between the iliac crest and the rib cage's lower limit. Blood pressure was measured following the recommendations of the European Society of Hypertension [28].

Ultrasound measurements were performed by a radiologist using a Toshiba Nemio 4D digital ultrasound and a 3.5 MHz convex transducer (Toshiba Medical Systems, Tustin, CA). The transducer was placed 1 cm above the umbilicus. Subcutaneous fat was defined as the distance between the skin and the external aspect of the abdominal rectus muscle, and visceral fat was the distance between the internal aspect of the same muscle and the anterior wall of the aorta [29]. The diagnosis of NAFLD was made in subjects with low or no alcohol consumption (<30 g/day) using the following criteria: increased liver echogenicity compared to the kidney, little or no visualization of the portal vessels and diaphragm, and sound attenuation [30]. An 8-hour fasting blood sample was taken to determine blood glucose, lipid profile, insulin, C-reactive protein, uric acid, and gamma-glutamyl transferase (GGT). A 75 g oral glucose tolerance test was performed. Glucose, triglycerides, total cholesterol, and insulin were measured on a Hitachi 917 *r* automated analyzer (Roche Diagnostics, Indianapolis, IN). Insulin resistance was measured by HOMA-IR index as follows = (fasting insulin (*µ*IU/ml) × fasting glucose (mmol/L)/22.5); the TyG index = Ln (triglycerides (mg/dL) × fasting glucose (mg/dL)/2), and the TG/HDL ratio = (triglycerides (mg/dL)/HDL-c (mg/dL)).

We used the diagnosis of metabolic syndrome based on the recommendations for Latin America with the population-adjusted criteria of the International Diabetes Federation (waist circumference ≥90 cm), together with two of the following criteria: fasting glucose >100 mg/dL (5.6 mmol/L), TG ≥ 150 mg/dL (1.70 mmol/L), high-density cholesterol <40 mg/dL (1.03 mmol/L), systolic blood pressure ≥130 or diastolic blood pressure ≥85 mmHg, or treatment of previously diagnosed arterial hypertension [31].

### 2.3. Statistical Analysis

Continuous variables were expressed as mean and standard deviation. The Wilcoxon rank-sum test was used to compare continuous variables between individuals with and without NAFLD. The values of HOMA-IR, TyG index, and TG/HDL ratio were divided into tertiles, and the prevalence of NAFLD was established for each one. The receiver operating characteristic (ROC) curves were used to determine the area under the curve (AUC) and the cutoff points of maximum sensitivity and specificity of IR markers (HOMA-IR, TyG, and TG/HDL) for diagnosing NAFLD. Univariate and multivariate logistic regression models were used to assess the associations between the IR markers (HOMA-IR, TyG, and TG/HDL) and NAFLD; we compared the risk of NAFLD in each tertile of IR markers with the lowest category of risk (reference group). These analyses were adjusted for potential confounders, such as age, BMI, waist circumference, hip circumference, and visceral and subcutaneous fat. The *R* software version 3.9.1 and the glm and pROC libraries were used.

## 3. Results

The anthropometric and biochemical characteristics of the 263 participants are given in Table 1. The NAFLD ultrasound criteria were met in 70 individuals (26.6%). Subjects with NAFLD had significantly higher body mass index, waist circumference, hip circumference, waist to hip ratio, subcutaneous fat, visceral fat, diastolic blood pressure, postload glycemia, triglycerides, HDL, basal insulin, uric acid, GGT, HOMA-IR, TyG index, and TG/HDL ratio. Furthermore, in subjects with NAFLD, 48.5% met the criteria for metabolic syndrome, unlike the subjects without NAFLD (13%). When comparing the three markers of IR between subjects with or without NAFLD, the individuals with NAFLD had significantly (*p* < 0.001) higher values of HOMA-IR (2.55 ± 1.36 vs. 1.51 ± 0.91), TyG (9.17 ± 0.53 vs. 8.7 ± 0.51), and TG/HDL (6.6 ± 4.54 vs. 3.52 ± 2.32).

**Figure 1:**
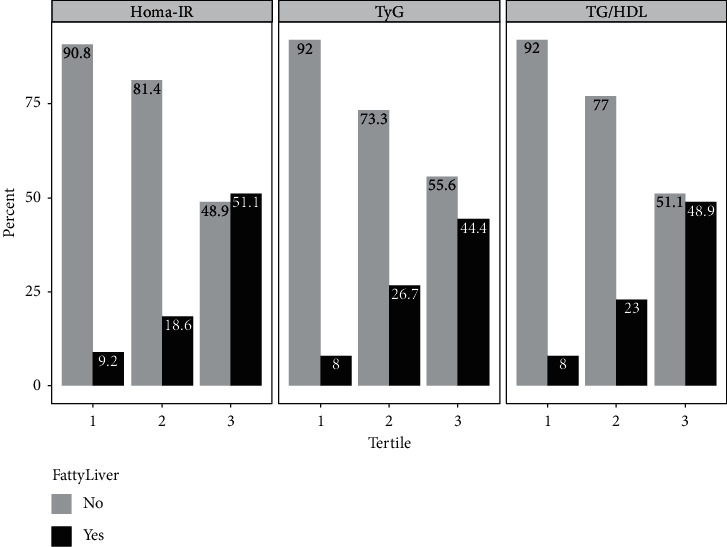
Prevalence of nonalcoholic fatty liver disease (NAFLD) across tertiles of insulin resistance markers (HOMA-IR, TyG, and TG/HDL).

The prevalence of NAFLD according to tertiles of IR markers is shown in Figure 1. It is observed that as the values of the HOMA-IR index, TyG index, and TG/HDL ratio increase, the prevalence of NAFLD increases; thus, subjects in the higher tertiles had a prevalence of NAFLD of 51.1%, 44.4%, and 48.9%, compared with subjects in tertile 1 9.2%, 8%, and 8%, respectively. Furthermore, the association between IR markers and NAFLD is given in Table 2. The risk of NAFLD increased with increasing tertiles of HOMA-IR, TyG, and TG/HDL. Compared with subjects in the lowest tertile, subjects in the highest tertile had four-fold the risk of NAFLD (HOMA-IR, OR = 4.08, 95% CI: 1.58–11.34; TyG, OR = 4.07, 95% CI: 1.54–11.88; TG/HDL, 4.05, 95% CI: 1.53–11.80). These associations were maintained after adjusting for potential confounders.

**Figure 2:**
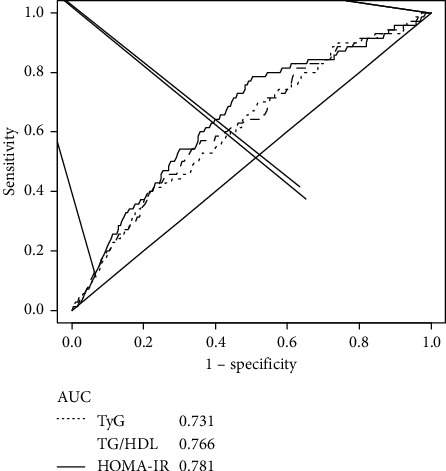
Receiver operating characteristic (ROC) curves and corresponding areas under the curve (AUC) for the diagnosis of nonalcoholic fatty liver disease (NAFLD).

Figure 2 shows the receiver operating characteristic (ROC) curves and the corresponding areas under the curve (AUC) of the three markers of IR to detect NAFLD. A HOMA-IR index value of 1.68 had an AUC of 0.781 (sensitivity 78% and specificity 71%). Similarly, a TyG index cutoff point of 8.92 showed an AUC of 0.731 (sensitivity 70%, specificity 66%), and a TG/HDL ratio cutoff point of 3.83 showed an AUC of 0.766 (sensitivity 71% and specificity 67%).

## 4. Discussion

Our study results show that the TyG index and the TG/HDL ratio, markers of IR, are useful for detecting NAFLD, with a diagnostic performance comparable to that of the HOMA-IR index. As IR plays a central pathogenic role in NAFLD development [32], indirect markers of IR could be the first step for early detection of this disease. Isokuortti et al. reported that a cutoff point of 1.9 for the HOMA index had 87% sensitivity and 79% specificity to detect the presence of NAFLD [33]. Previously, we have reported that the HOMA-IR index has a sensitivity of 72% and specificity of 73% to detect NAFLD [34]. However, its calculation requires the measurement of insulin levels, which reduces its potential for routine use in clinical practice both due to its wide variability and financial cost, the latter even more of a limitation in less well-resourced healthcare systems. Importantly, the present results showed that the TyG index and the TG/HDL ratio have a sensitivity and specificity near 70% for NAFLD detection, making them comparable to the HOMA-IR index. This performance is similar to previous studies on Asian populations [17, 18]. In a study of 4,986 Korean subjects followed in a primary prevention program, Lee et al. reported that as the TyG index increases (comparing Q2 vs. Q4), the risk of NAFLD increases (OR = 1.93 vs. 2.94, respectively *p* < 0.01); the AUC to detect NAFLD for the TyG index was 0.716 and for the HOMA-IR index was 0.67 [19]. Furthermore, a large cohort study in the Chinese population diagnosed with NAFLD (*n* = 11,424) demonstrated that after 21 months of follow-up, the TyG index could be a helpful indicator of NAFLD progression [35]. These results could be explained by the fact that the TyG index reflects the pathophysiological mechanism of glucolipotoxicity in the genesis of IR [36], since it reveals the role of ectopic fat deposits that cause a decrease in glucose transport at muscular and liver levels, generating a lack of inhibition of hepatic glucose production, glycogen synthesis, and increased hepatic lipogenesis [37, 38].

Guo et al. [14] evaluated the role of the TyG index as a predictor of NAFLD in 4,784 Chinese subjects using an ultrasonographic diagnosis and found that NAFLD prevalence increased across quartiles of TyG (prevalence of 30.9%, 53.3%, 71.7%, and 86.4% in Q1–Q4, *p* < 0.01). Applying a cutoff point of 8.7, the AUC was 0.761, with a sensitivity of 70.6% and a specificity of 69.1%.

Tarantino et al. evaluated the presence of NAFLD by the TyG index (cutoff of 0.59) and IR assessed by the same TyG index (cutoff 4.68) and the TG/HDL ratio (cutoff 2.197) in 204 nonmetastatic bladder cancer (BCa) patients and 50 subjects with no BCa but with bladder diseases (no Ca BD), finding that TyG index predicted NAFLD in both groups(*p*=0.0001) and found a greater proportion of IR (47%) in the BCa group than in no Ca BD one (37%) [39]. Several studies have shown that TyG values above 8.5 correctly identify subjects with NAFLD, which aligns with our findings [15, 19, 20]. In contrast, a study of 50 asymptomatic women reported a value of 4.58 to define NAFLD [38], a finding which may be related to differences in the method of calculating the TyG index. Specifically, some authors divide the result of the logarithm of the product of triglycerides and glucose by two [20, 21, 40], while others apply the logarithm to the result of the division by 2 of the triglyceride product glucose [19]. The current lack of standardization of the TyG index formula could also limit its clinical use.

The usefulness of the TG/HDL ratio in detecting NAFLD is also well established. For example, in a study conducted on 18,061 apparently healthy Chinese individuals, the prevalence of NAFLD was 24.8% and was independently associated with TG/HDL ratio. The prevalence of NAFLD progressively increased across the quartiles of TG/HDL (4.9, 14.1, 26.8, and 53.5%, respectively, *p* < 0.001), compared to quartile 1 [41]. However, the present analysis is to our knowledge the first study to compare the diagnostic performance of this index with that of the HOMA-IR index for NAFLD. The similar performance of TyG and TG/HDL indexes means they could be considered effective, noninvasive, and low-cost screening tools easily implemented as the initial step at different healthcare levels of the clinical practice to identify subjects with NAFLD. They also as markers of IR are associated with other cardiometabolic alterations related to IR, such as cardiovascular disease, diabetes, and hypertension. For example, in a cohort of 6,078 subjects from China followed for six years, those in quartiles 3 and 4 of the TyG index had a higher risk of cardiovascular events compared to quartile 1 (HR = 1.33, 95% CI 1.05–1.68; HR = 1.72, 95% CI 1.37–2.16, respectively) [23]. In a nine-year prospective study in a population of 4,686 Chinese subjects, Zheng Mao established an association with the incidence of hypertension. The overall incidence of hypertension was 43.7%, increasing across the quartiles of the TyG index from 28.5% in Q1, 36.9 in Q2, 49.2 in Q3, and 59.8% in Q(*p* < 0.001)[24].

The present study has some limitations, including the sample size, which can be considered small. Second, the studied population corresponds to men from the Air Force, who despite being homogeneous, are with limited variability in other risk factors for NAFLD (such as excess alcohol intake), and other dietary and physical activity patterns were not evaluated. It could be assumed that the habits of the Air Force personnel are not generalizable to other populations. Third, the diagnosis of NAFLD was made by ultrasonography, which is an operator-dependent test, and its sensitivity for the diagnosis of NAFLD may be lower in people with obesity. However, it is recently shown that the TyG index is associated with histopathological findings of NAFLD in liver biopsies [42].

## 5. Conclusions

The present study demonstrates the effectiveness of the TyG index and the TG/HDL ratio, with cutoff points of 8.92 and 3.82, respectively, as clinical tools to identify individuals with NAFLD in a Hispanic male population, with a similar performance as HOMA-IR and lower cost. These findings are important as the identification of NAFLD by imaging is impractical as a screening tool at a population level. We propose that the TyG index or the TG/HDL ratio should be used instead of insulin measurement and HOMA-IR calculation as the first clinical step in identifying individuals with probable NAFLD, as well as other pathologies associated with IR. Further studies should confirm this in other populations, and prospective studies are needed to establish the relationship between these markers and the development of cardiometabolic alterations.

## Figures and Tables

**Table 1:** Baseline characteristics according to the presence of nonalcoholic fatty liver disease (NAFLD).

	Subjects with NAFLD (*n* = 70)	Subjects without NAFLD (*n* = 193)	*P* value^*∗*^
Age (years)	39.31 (±5.31)	38.35 (±5.97)	0.13
BMI (kg/m^2^)	26.88 (±2.56)	23.92 (±2.52)	<0.001
Waist circumference (cm)	94.4 (±5.8)	86.8 (±6.7)	<0.001
Hip circumference (cm)	97.9 (±9.3)	92.5 (±5.1)	<0.001
Waist-to-hip ratio	0.97 (±0.06)	0.95 (±0.05)	<0.001
Subcutaneous fat (mm)	19.4 (±6.7)	15.2 (±4.7)	<0.001
Visceral fat (mm)	40.8 (±12.7)	27.4 (±12.2)	<0.001
Systolic blood pressure (mmHg)	116.19 (±14.43)	113.23(13.97)	0.142
Diastolic blood pressure (mmHg)	74.01 (±9.5)	71.2 (±10.0)	0.042
Fasting glycemia (mg/dl)	92.5 (±8.01)	91.03 (±8.35)	0.182
Post 75 g OGTT glycemia (mg/dl)	91.23 (±27.54)	81.16 (±20.4)	0.006
TG (mg/dl)	238 (±136.6)	152 (±78.76)	<0.001
HDL (mg/dl)	38.73 (±7.46)	47.57 (±11.5)	<0.001
Insulin (*µ*IU/ml)	11.01 (±5.13)	6.64 (±3.64)	<0.001
Uric acid (mg/dl)	7.01 (1.2)	6.38 (1.29)	<0.001
GGT (IU/l)	67.1 (81.57)	36.62 (32.51)	0.003
HOMA-IR	2.55 (±1.36)	1.51 (±0.91)	<0.001
TyG index	9.17 (±0.53)	8.72 (±0.51)	<0.001
TG/HDL ratio	6.6 (±4.54)	3.52 (±2.32)	<0.001

BMI, body mass index; OGTT, oral glucose tolerance test; TG, triglycerides; HDL, high-density lipoprotein; GGT, gamma-glutamyl transferase; HOMA-IR, the homeostasis model assessment index; TyG, triglycerides/glucose; TG/HDL, triglycerides/high-density lipoprotein.

**Table 2:** Association between insulin resistance markers (HOMA-IR, TyG, and TG/HDL) and nonalcoholic fatty liver disease (NAFLD).

IR markers	Model 1^§^			Model 2^*∗*^		
	OR	IC 95%	*P* value	OR	IC 95%	*P* value
HOMA-IR						
T1 (0.24–1.23)	Ref.					
T2 (1.24–1.94)	2.26	0.93–5.87	0.079	1.79	0.66–5.15	0.262
T3 (1.95–9.24)	10.32	4.69–25.42	**<0.001**	4.08	1.58–11.34	**0.004**
TyG index						
T1 (7.21–8.58)	Ref.					
T2 (8.59–9.08)	4.17	1.76–11.09	**0.002**	3.17	1.18–9.42	**0.020**
T3 (9.09–10.87)	9.14	4.01–23.74	**<0.001**	4.07	1.54–11.88	**0.003**
TG/HDL ratio						
T1 (0.48–2.57)	Ref.					
T2 (2.58–4.44)	3.41	1.42–9.15	**0.008**	1.69	0.60–5.12	0.331
T3 (4.45–32.1)	10.92	4.79–28.39	**<0.001**	4.05	1.53–11.80	**0.007**

IR, insulin resistance; HOMA-IR, the homeostasis model assessment index; TyG, triglycerides/glucose; TG/HDL, triglycerides/high-density lipoprotein. ^§^Unadjusted model. ^*∗*^Adjusted model for age, BMI, waist circumference, hip circumference, visceral, and subcutaneous fat.

## Data Availability

The data used to support this study are not publicly available but are available from the corresponding author upon request.
